# Draft Genome Sequence of *Pseudomonas putida* BW11M1, a Banana Rhizosphere Isolate with a Diversified Antimicrobial Armamentarium

**DOI:** 10.1128/genomeA.00251-16

**Published:** 2016-04-14

**Authors:** Maarten G. K. Ghequire, Toon Swings, Jan Michiels, Harald Gross, René De Mot

**Affiliations:** a Centre of Microbial and Plant Genetics, KU Leuven, Heverlee, Belgium; b Department of Pharmaceutical Biology, Pharmaceutical Institute, University of Tübingen, Tübingen, Germany

## Abstract

In this study, we report the draft genome of *Pseudomonas putida* BW11M1, a banana rhizosphere isolate producing various antimicrobial compounds, including a lectin-like bacteriocin, an R-type tailocin, the cyclic lipopeptide xantholysin, and the fatty acid–derived pseudopyronine.

## GENOME ANNOUNCEMENT

*Pseudomonas* is a metabolically versatile genus and its species produce a plethora of antimicrobials, which may target related and unrelated microorganisms to interfere with their proliferation. Such antibiosis can be mediated by different types of secondary metabolites (1), as well as by peptides or proteins (2). *Pseudomonas putida* BW11M1, a banana rhizosphere isolate from Sri Lankan wetlands (3), produces a variety of such molecules. This strain secretes a novel type of bacteriocin (4), which, unlike the classical toxin-immunity pairs (5, 6), does not require an immunity partner. Structurally related to plant lectins (7), this pseudomonad-targeting bacteriotoxic protein is predominantly occurring in plant-associated bacteria but is relatively rare in the opportunistic human pathogen *Pseudomonas aeruginosa* (8, 9). The bacteriocin complement of *P. putida* BW11M1 also includes a multiprotein complex evolutionary related to phage tails (10), and hence is referred to as a tailocin (11). The BW11M1 tailocin bears similarity with the R pyocin of *P. aeruginosa*, a contractile nanodevice puncturing target cells (12). The ability of *P. putida* BW11M1 to kill competitors is not restricted to other pseudomonad targets and also involves different types of secondary metabolites. The nonribosomally synthesized cyclic lipopeptide xantholysin inhibits the growth of several xanthomonads and fungi (13). A single BW11M1 enzyme generates the antibiotic pseudopyronine, which is mainly active against Gram-positive bacteria. This involves a one-step condensation fusing two fatty acids provided by primary metabolism (14), similar to the biosynthesis by the insect pathogen *Photorhabdus luminescens* of the signaling molecule photopyrone (15).

High-quality genomic DNA (Gentra Puregene Yeast/Bact. Kit, Qiagen) was subjected to 100-cycle paired-end massive parallel sequencing with the Illumina HiSeq2000 (GeneCore, EMBL, Heidelberg). CLC Genomics Workbench version 6.5.1 (https://www.qiagenbioinformatics.com) was used for analysis of the sequences. Following quality assessment of the raw data, reads were trimmed using quality scores of the individual bases. The quality limit was set to 0.01, and the maximum allowed number of ambiguous bases was set to 2. Reads shorter than 15 bases were discarded from the set. After trimming, the average length of the remaining reads was 81.3 bp. These reads were used for *de novo* assembly using the CLC Assembly Cell version 4.0 algorithm. This tool utilizes de Bruijn graphs for analysis of overlapping reads, which is often used for analyzing short-read sequencing data (16, 17). Assembly of 11,901,024 reads (167-fold median coverage) yielded 65 contigs with an *N*_50_ value of 225,130 bp. The average contig length is 83,952 bp, and the largest contig is 524,553 bp. The total assembled length is 5,456,879 bp, with a G+C content of 64.6%.

Analysis of the BW11M1 genomic sequence predicts the capacity to produce additional secondary metabolites likely involved in antibiosis, including hydrogen cyanide (18), a toxoflavin-related metabolite (19), and a nematode-deterring factor (20). Ribosomally encoded exoproteins with antagonistic potential include type VI secretion substrates, Rhs proteins, a contact-dependent inhibition toxin (2), and chitinolytic enzymes (21).

### Nucleotide sequence accession numbers.

This whole-genome shotgun project has been deposited at DDBJ/ENA/GenBank under the accession number LSLE00000000. The version described in this paper is the first version, LSLE01000000.
